# Intra thoracic extra pulmonary hydatidosis: prognosis and outcomes of 8 operated patients

**DOI:** 10.1186/s13019-023-02115-6

**Published:** 2023-01-15

**Authors:** A. Machboua, F. Z. Elhani, R. Marouf

**Affiliations:** 1 Department of Cardiothoracic Surgery, Mohammed VI University Hospital, Oujda, Morocco; 2 Laboratory of Anatomy, Microsurgery and Experimental Surgery and Medical Simulation, faculty of medecine and pharmacy, University Mohamed I, Oujda, Morocco

**Keywords:** Hydatid cyst, Intrathoracic extra pulmonary location, Surgery, Complications, Prognosis

## Abstract

Hydatid cyst disease is a parasitic disease known from the times of hippocrates, and is still endemic in our country Morocco among others, affecting mainly the liver and lungs, while intra thoracic extra pulmonary location remains a rare entity of the disease. In our department of thoracic surgery, Mohamed VI University Hospital, Oujda, Morocco, we operated 92 patients for thoracic hydatid cyst in the period between January 2016 and December 2021, 8 patients of this group had exclusive extra pulmonary location of the hydatid cyst, epidemiological and clinical data were recorded for the 8 patients (5 men, 3 women). The mean age was 40.3 years, all patients presented mainly with chest pain, dyspnea and cough. The locations of the hydatid cysts were chest wall, pericardium, pleural space and diaphragm. The hydatid cysts were removed via extirpation technique through thoracotomy in all patients. The average duration of hospitalization was 7 days. Postoperative complications consisted of atelectasis in one patient, parietal hematoma in another, and surinfection with pleuro-cutaneous fistula and chronic neurological chest pain in one patient. No deaths were noted in our series.

## Backgrounds

Intra thoracic yet extra pulmonary cyst was defined as a cyst that was found in intrathoracic extra pulmonary tissues, with no involvement of the pulmonary parenchyma and no transmission of the disease from abdomen to the thorax [1], We report 8 cases of extra pulmonary intra thoracic hydatidosis, where we aim to delineate our clinical and surgical experience in managing intra thoracic yet extra pulmonary hydatid cysts and descript the prognosis of this rare localization.

## Patients and methods

We reviewed clinical records for 92 patients who underwent surgical treatment in our department for thoracic hydatidosis between January 2016 and December 2021, amidst 92 patients 8 had intra thoracic extra pulmonary hydatid cysts without pulmonary association, this last group constituted our case series.

We listed the age, gender, history, notion of hydatid contagion, symptoms, localization of intrathoracic extra pulmonary hydatid cysts and surgical treatment procedures, complications, morbidity, mortality, hospitalization time after operation and recurrence for each patient (Table 1). Diagnostic tools consisted mainly of chest radiography and computed tomography (CT) of the chest and upper abdomen for all patients, magnetic resonance imaging (MRI) for 3 patients, echocardiography for 1 patient and abdominal ultrasound in all patients in addition to hydatid serology.

**Table 1:** Patient’s clinical, radiological and surgical records

Patients	Age (years), gender	Antecedents	Symptomatology	Location and appearance on radiology	Serology	Surgical procedure	Evolution, complications
Patient 1	19, man	Rural origin	Chest pain, cough, dyspnea	Pleural: Enormous hydatid lesion of the left pleural cavity, with pulmonary passive atelectasis	Negative	Total excision under posterolateral thoracotomy with pulmonary decortication	Discharged à J6 postopératif day No recurrence after 3 years
Patient 2	26, man	Rural origin	Left chest and back pain, lower limb paresthesia	Costo-vertebral: Cystic-like formation with fluid content and multiple vesicles; occupying the left costovertebral angle with lysis of the posterior arch of the 7th, 8th and 9th ribs., with foraminal extension D7-D8, with spinal cord compression (Fig. 3)	Positive	Monobloc resection of the cyst and posterior arches of 4 ribs and vertebral bodies of D8-9 with osteosynthesis of the spine under à large posterolateral thoracotomy	Pleurocutaneos fistula, with disabling parietal and neurological pain and a disorder of the statics of the shoulder girdle No reccurence of hydatid disease after 2 years
Patient 3	42, woman	Operated for liver hydatid cyst	Chest pain, dyspnea	Parietal: multiple right parietal and diaphragmatic cystic lesions	Not done	Total excision of all the cyst under à posterolateral thoracotomy	No complications and no recurrence after 1 year
Patient 4	32, woman	Operated for liver hydatid cyst	Left back and chest pain, dyspnea	Costo-vertebral: left postero-basal multi-vesicular cyst of the costovertebral splint at the expense of the 7th and 8th ribs with costal lysis	Positive	Total excision of the cyst and affected ribs without parietal prosthesis under a posterolateral thoracotomy	No recuernce and no complications
Patient 5	28, man	Operated for liver and pulmonary hydatids cysts	Chest pain, dyspnea	Costal: right latero-basal thoracic cyst, with invasion of the soft tissues opposite and lysis of the lateral arch of the 8th rib, without extension to the liver (Fig. 1)	Not done	Total excision of the cyst and affected ribs without parietal prosthesis under a direct approach	No recuernce and no complications
Patient 6	57, man	Rural origin	Chest pain, parietal swelling	Costal: right large circumscribed hypo dense mass localized between the 6th and 7th rib, adherent to the lung causing destruction of the ribs with scarce micro calcifications	Positive	Total excision of the cyst and affected ribs without parietal prosthesis under a posterolateral thoracotomy	No recurrence, no complications
Patient 7	57, man	Operated for liver hydatid cyst	Palpitations, dyspnea	Pericardial: pericardial right hydatid lesion without involvement of the atrial wall (Fig. 2)	positive	Total excision of the cyst and the affected pericardic without paericardic pach under an anterior thoracotomy	No complications, no recurence
Patient 8	63, woman	Operated for liver and pulmonary hydatid cyst	Chest pain	Costal: well-limited cystic mass in the pleural cavity opposite the right upper lobe, measuring 50 mm in length, invading the posterior arch of the 4th, 5th and 6th sides and the 4th intercostal space	Not done	Total excision of the cyst under a posterolateral thoracotomy	Accidental peropérative lésiosn of subclavier artery

## Results

**Fig. 1:**
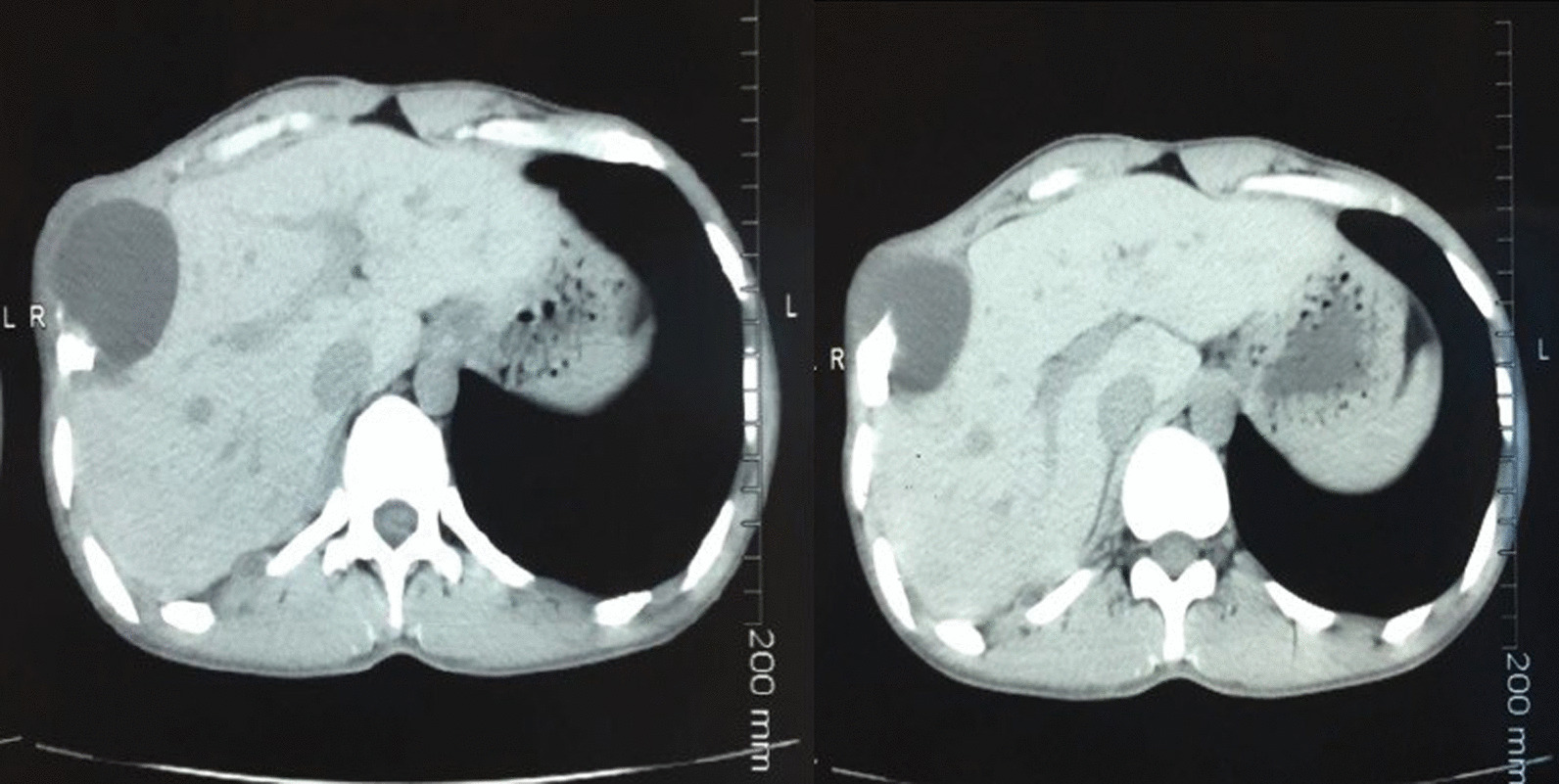
Thoraco-abdominal CT scan showing an a latero-basal thoracic hydatid cyst, measuring 5 cm in large diameter, invading the soft parts and the lateral arch of the 8th rib, without hepatic impairment

**Fig. 2:**
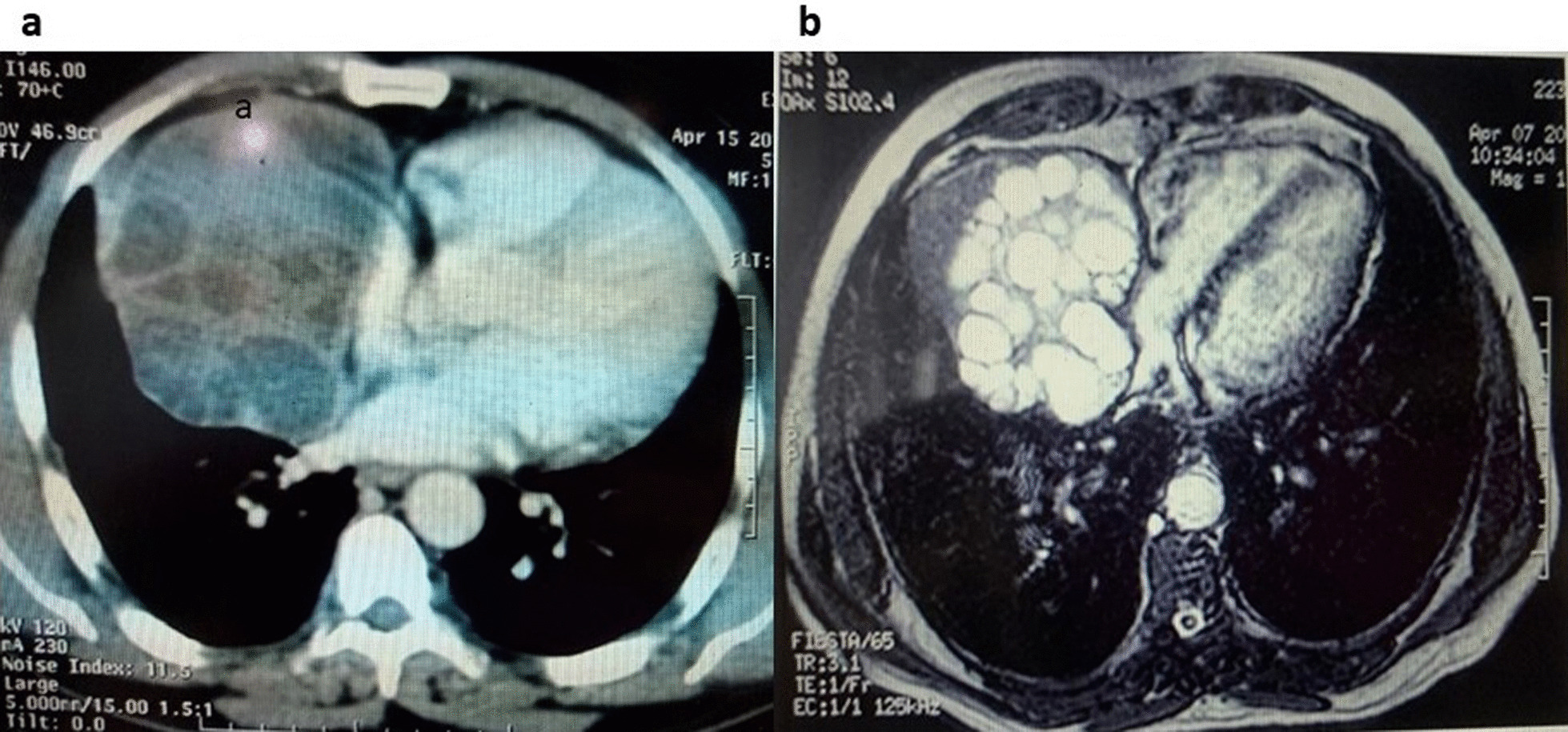
**a** CT scan established a 11 × 7 cm heterogeneous cystic lesion with unclear boundaries with the pericardium compressing the right atrium and the inferior vena cava. **b** MRI T2-weighted image, showing the pericardial nature of the lesion without involvement of the atrial wall; the lesion is hyper intense on T2 and hypo intense on T1-weighted images

**Fig. 3:**
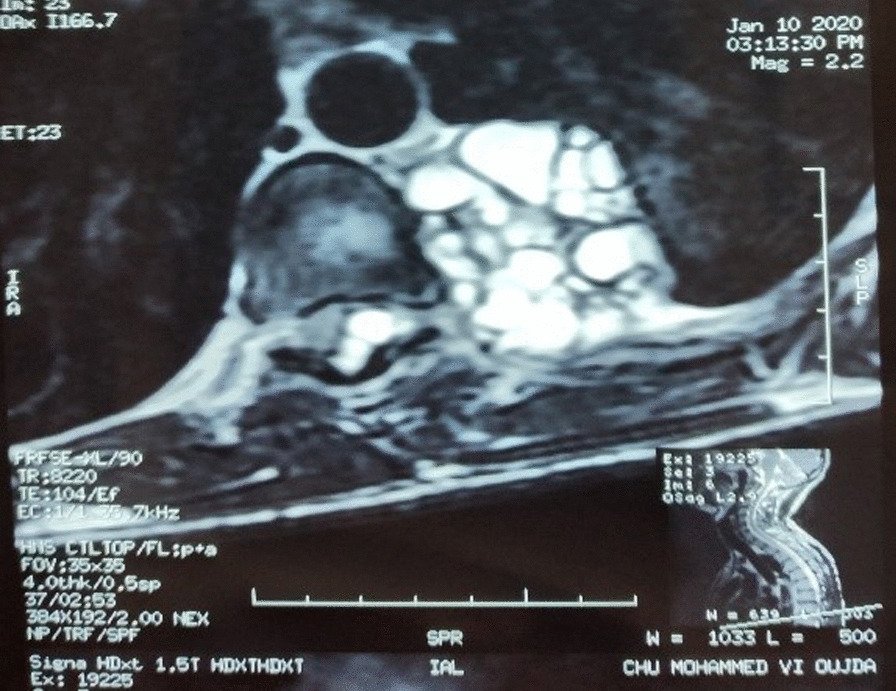
MRI showing costovertebral hydatid cyst with the presence of endocanal vesicles suggesting an invasion of the bone marrow with risk of medullary compression

This study group comprised of 5 men and 3 women with a gender ratio of 1.66, an average age of 40.5 ranging from 19 to 63 years. A notion of contact with dogs was found in 7 patients (87.5%), 5 out of 8 patients had antecedents of prior hydatid cyst of either pulmonary or hepatic location or both, all patients were symptomatic and presented mainly with chest pain, dyspnea, and cough, no hemoptysis or hydatidoptysis were observed in our patients. All 8 patients benefited from chest X ray and chest CT (Figs. 1, 2a), while MRI was performed in 3 cases in order to delineate precise contiguity to adjacent tissues; in 2 Patients with costo-vertebral hydatid cyst, in one of them, MRI demonstrated medullary invasion with presence of hydatid vesicles within the medullary canal (Fig. 3), while in one patient with pericardial hydatid cyst MRI had shown pericardial nature of the lesion without involvement of the atrial wall; the lesion was hyper-intense on axial T2-weighted images and hypo-intense on axial and coronal T1-weighted images (Fig. 2b). Abdominal ultrasound was performed in all patients and found no abdominal associated locations, chest ultrasound was performed in 1 patient with pericardial location in order to determine contiguity to the pericardium and showed a mass-like appearance with heterogeneous echogenicity in the right atrial wall, which was initially thought to be a primary atrial myxoma, compressing the right atrium and inferior vena cava. Hydatid serology using ELISA haemaglutination had been performed in 5 patients, and rendered true positive results in 4 cases.

Regarding the cysts locations, we were able to identify 4 parietal hydatid cysts (3 costal, one without costal damage), in 4 different patients; in one of them the parietal cyst was associated with 2 other diaphragmatic cysts, 2 costo-vertebral hydatid cysts, 1 pleural hydatid cyst, and 1 pericardial hydatid cyst (Table 1).

**Fig. 4:**
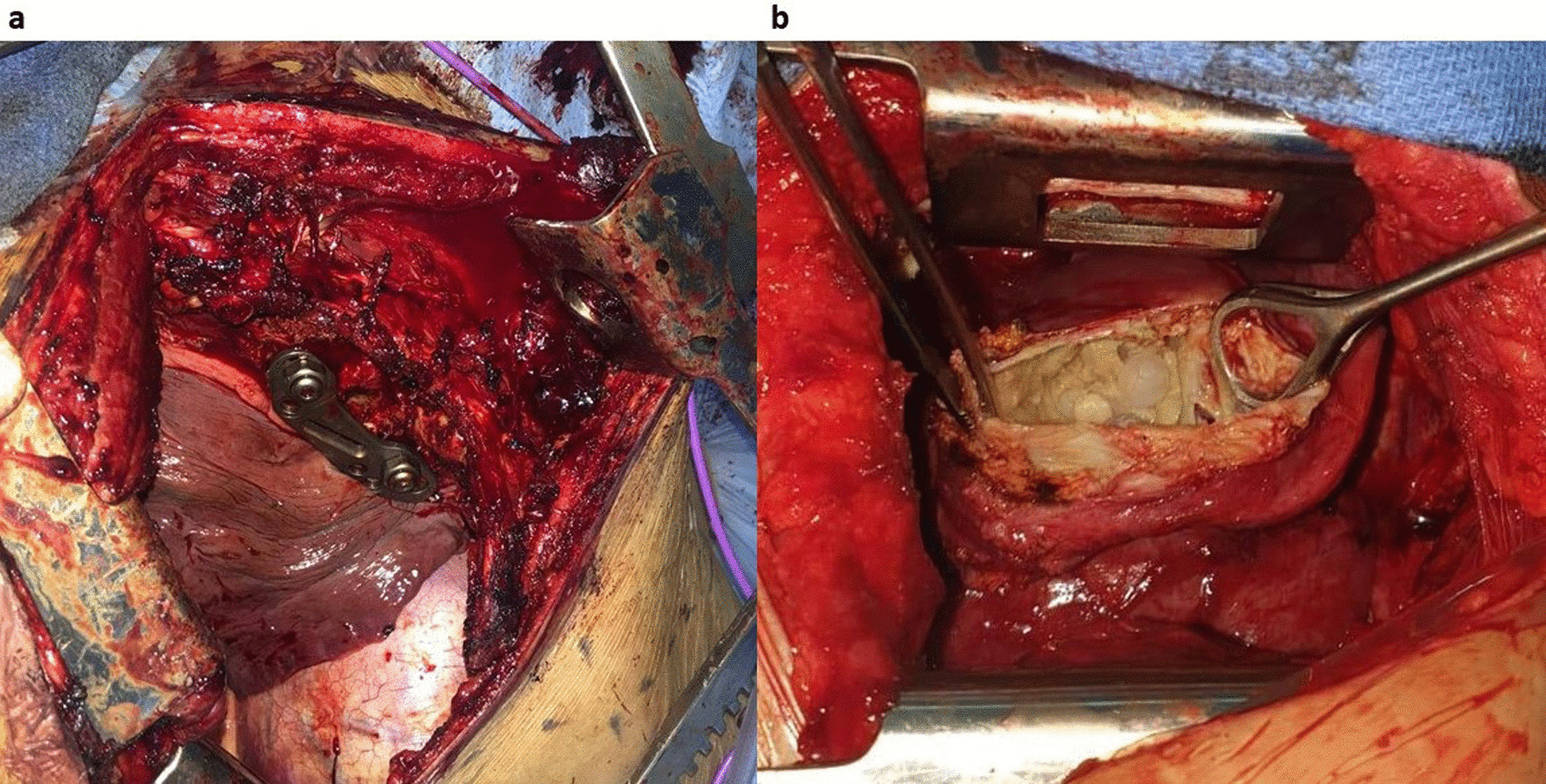
Perioperative view, **a** showing à result after excision of capsulated cystic mass in left paravertebral thoracic region and implant stabilization of the spinal column, **b** perioperative view of a pericardial hydatid cyst

**Fig. 5:**
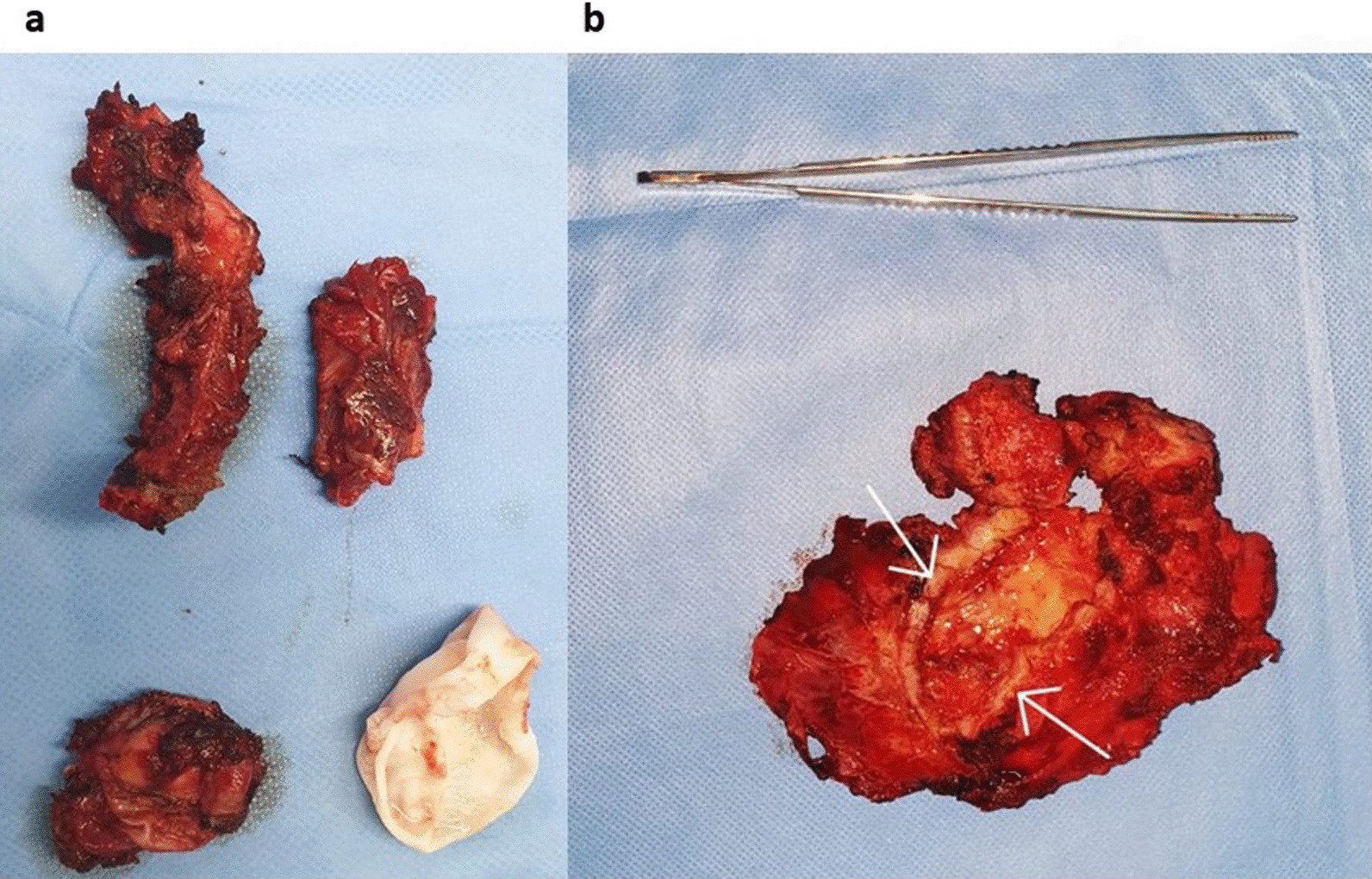
Photos of the surgical specimens, showing the complete resection of the lesions: **a** costal resection removing the hydatid cyst, **b** hydatid cyst located between the visceral and parietal pleura (the limits of the cyst capsule are indicated by the white arrows)

Radical treatment was of choice in all our patients as more conservative techniques might invite into spillage and recurrences, surgical treatment was performed through postero-lateral thoracotomy in 6 patients (costovertebral, costal, diaphragmatic and pleural), anterolateral thoracotomy in 1 patient carrying pericardial hydatid cyst, and a direct approach to the lesion in 1 patient. All patients benefited from total resection of the lesion with peri-cystectomy and lavage with hypertonic solution. In the costovertebral hydatid cyst, the excision of capsulated cystic mass in left paravertebral thoracic region was carried out with decompression of the spinal cord, and implant stabilization of the spinal column (Fig. 4a), for pericardial localization a monobloc total resection of the lesion and the surrounding pericardial tissue was carried out without impairment of the pericardium thus no reconstruction techniques were performed (Fig. 4b), diaphragmatic hydatid cyst was seen in one patient, and total resection of the cyst was performed along with debridement and curettage without impairment of the diaphragm thus needing no suturing. Costal localizations (4 patients) were treated through total excision of the lesion along with rib resection, without any parietal reconstruction due to the small size of the lesions, while pleural localization was treated through excision of the cyst and affected pleural tissue as it caused pleural thickening due to inflammation (Fig. 5a, b). Although the great use of diagnostic tools already mentioned yet diagnosis was mainly attained intra operatively.

For intraoperative complications, an accidental injury of the subclavian artery requiring its clamping for repair was noted in one patient with a parietal hydatid cyst adherent to the subclavian pedicle. Immediate post-operative complications were usual and were noted in 2 patients, one case of atelectasis in a patient with pleural localization, and one parietal hematoma in another patient requiring revision under thoracoscopy, patients had an average hospitalization duration of 7 days, standard deviation (4.14 days), all patients received antiparasitic treatment with Albendazol 400 mg/day for 6 months after surgical resection. No deaths were noted in our case series.

Long term complications through follow up of 8–60 months and an average of 34 months, revealed no recurrence in all 8 patients. Although a major complication was noted consisting in the persistence of a pleuro-cutaneous fistula without hydatid recurrence in a patient treated for costovertebral hydatid cyst with osteosynthesis of the spine, with disabling parietal and neurological pain and a disorder of the statics of the shoulder girdle; all of which caused a social and functional repercussion for the patient, who was obliged to give up his job as a hairdresser, with recourse to antidepressant treatment and functional rehabilitation.

## Discussion

Hydatid cyst is caused by the parasite *Echinococcus granulosus*. The adult worm resides in the jejunum of dogs and other canines. It produces eggs that are passed in the feces. Eggs ingested by intermediate hosts such as cows and sheep, and accidentally humans, the latter reach the duodenum and produce embryos, which pass into the portal circulation. At the site most of these embryos are trapped in the liver, but some pass through and reach other organs, developing into hydatid cysts. The liver is the most common site, followed by the lung. All other organs can be affected, but this is rare, including extra-lung intrathoracic organs (pleura, mediastinum, chest wall, pericardium, diaphragm) [2].

Thoracic extra pulmonary hydatidosis localization is considered a rare entity of hydatid disease, moreover when it is not associated to pulmonary or hepatic localization. In our series, we have noted predominance of chest wall localization with a staggering 75% (6 patients), while in Dakak et al. series [3] mediastinal localization was predominant with 42% (14 out of 33 patients), in Oguzkaya et al. series [4] pleural localization came in first with 72.7% (16 out of 22 patients). In extra pulmonary intrathoracic hydatid cyst authors have suggested a new theory of hydatid dissemination via ascending diaphragmatic lymphatic drainage along the internal mammary nodes anteriorly, and the intercostal nodes posteriorly [5]. A hematological dissemination may be possible via an arterial branch of the thoracic aorta such as intercostal or diaphragmatic arteries [6].

In our series, patients presented mainly with chest pain, and dyspnea. Other authors reported same symptoms in addition to syncope and dysphagia due to compression of mediastinal entities in mediastinal localizations; which was not observed in our series as we didn’t have any mediastinal localization. The clinical symptomatology is not specific; all types of symptoms related to a compression of the surrounding organs can be noted, even an incidental discovery is possible. CT is of great use; it has an essential role in the diagnosis; particularly in the presence of daughter vesicles; as the presence of a multi-vesicular form, almost always reflects an extra-pulmonary localization of the hydatid cyst [7]. CT shows classically a roughly rounded, hypodense, homogeneous, partitioned or heterogeneous mass depending on the evolutionary stage of the cyst, and it helps to plan the surgical management and the adapted approach, while specifying its exact location, its size, its anatomical relations with neighboring organs and the presence of any associated lesions. MRI came in handy especially for mediastinal and costo-vertebral localization as to determine with precision medullary impairment and medullary compression requiring emergency surgical treatment, causing paraplegia and endangering functional prognosis of the patient, although being benign this particular entity of hydatid disease is entitled ‘white cancer’ because it requires à large monobloc excision with healthy margins [8].

In pericardial localization of the disease, right cardio phrenic location is by far the most popular [9], which is the case in our series, as we reported one case of pericardial hydatid cyst that was explored through echocardiography, making it possible to determine the cystic nature of the lesion and eliminate differential diagnosis such as myxoma, fibroma or intra cardiac thrombosis.

Although the great use of paraclinical diagnostic tools; diagnosis of thoracic extra pulmonary hydatidosis remains difficult due to multiple differential diagnosis such as mediastinal dermoid cysts, bronchogenic cysts or pleuro pericardial cysts. Therefore, positive diagnosis is mainly attained through surgical exploration.

As an endemic country, thoracic extra pulmonary hydatidosis should be suspected in patients with antecedent of hydatid cyst, concomitant hydatid cyst, or positive hydatid serology [7].

The complete surgical resection of all infected tissue is the gold standard treatment, it should be carried out through a large approach such as posterolateral thoracotomy for optimum control of the lesion to guarantee total extirpation of the lesion in order to avoid spillage and recidivism, or a median sternotomy for anterior mediastinal, pericardial cyst as reported by Kabiri et al. [10].

In diaphragmatic localizations excision of the lesion may at times need reconstruction with prosthetic mesh or simple suture if no important defects were found [11]. In costal localization partial or total rib resections are needed in addition to excision of adjacent affected tissues [12, 13], while in mediastinal localization, extensive resection is to be avoided when the progression of dissection is impossible or dangerous due to intimate adhesions to vital entities, as reported by Traibi et al. [14], in their series partial péricystectomy was carried out in 5 patients, so the aim here is radical removal of germinate membrane and pericyst when it is possible, because of complications for mediastinal locations can be serious.

Radical treatment is the standard in thoracic extra pulmonary hydatidosis, the goal being total resection of the cyst, the pericyst, and the adjacent affected tissues in order to avoid any recurrence of the disease [15].

Thoracic extra pulmonary hydatidosis is a benign disease yet, the majority of authors consider it as a localized malignancy tumor known by the term ‘white cancer ‘which explains the radical therapeutic attitude towards it and large excision margins [8, 9, 12], although there is a risk of complications in large resections. This particular approach was carried out in one of our patients carrying a costovertebral cyst; where excision of capsulated cystic mass with resection of 4 posterior rib arcs and vertebral bodies, in left paravertebral region was carried out, with decompression of the spinal cord by laminectomy, in addition to implant stabilization of the spinal column.

The therapeutic management of this rare entity of hydatid cyst is essentially based on radical and complete surgical resection of all lesions and damaged tissues, without conservation to avoid the risk of recurrence, and the association of an antiparasitic medical treatment for 6 months or more based on azoles to consolidate the benefit of the surgery.

The prognosis of this pathology is overall good, in our series, no per or postoperative mortalities were noted, major parietal resections, especially in costovertebral locations, can be responsible for chronic complications such as disabling neurological pain, instability of the chest wall and possible deformation, along with higher risk of infection in the case of the utilization of a prosthesis, as it is the case of our patient who had costovertebral location which required à large resection of the chest wall.

## Conclusion

Extra-pulmonary intrathoracic hydatid cyst is a rare entity requiring radical surgical removal of all damaged tissues with large healthy margins, associated with medical treatment with Albendazol.

## Data Availability

The data used belongs to the archive of our service, access to it is a possible.

## References

[1] Gursoy S, Ucvet A, Tozum H (2009). Primary intrathoracic extrapulmonary hydatid cysts: analysis of 14 patients with a rare clinical entity. Tex Heart Inst J.

[2] Gopivallabha MM, Madhusudhan M, Singh AK (2020). Hydatid cyst causing diaphragmatic palsy. Braz J Cardiovasc Surg.

[3] Dakak M, Yucel O, Kavakli K (2009). Intrathoracic extrapulmonary hydatid cysts: review of 33 cases. Trak Univ Tip Falk Derg.

[4] Oguzkaya F, Akçali Y, Kahraman C (1997). Unusually located hydatid cysts: intrathoracic but extrapulmonary. Ann Thorac Surg.

[5] Isitmangil T, Toker A, Sebit S (2003). A novel terminology and dissemination theory for a subgroup of intrathoracic extrapulmonary hydatid cysts. Med Hypotheses.

[6] Farah A, José Manuel R, Luis G (2017). Extrapulmonar and extrahepatic hydatidosis. Cir Cir.

[7] Bin Saeedan M, Musallam Aljohani I, Ali Alghofaily K (2020). Thoracic hydatid disease: a radiologic review of unusual cases. World J Clin Cases.

[8] Marouf R (2014). Hydatid cyst in costo-vertebral location. Pan Afr Med J.

[9] Marouf R, Alloubi I (2019). Hydatid cyst of the pericardium mimicking a right atrial myxoma. Indian J Thorac Cardiovasc Surg.

[10] Kabiri E, El Hammoumi M, Hairis M (2020). Kyste hydatique médiastinal calcifié. Acta Chir Belg.

[11] Salih AM, Kakamad FH, Rauf GM (2016). Isolated hydatid cyst of the diaphragm, a case report. Int J Surg Case Rep.

[12] Marouf R, Alloubi I. Kyste hydatique costal : à propos d’un cas et revue de la littérature. J Chir Thorac Cardio-Vasc. 2019;23(1).

[13] Roman A, Georgiu C, Nicolau D (2015). Cystic hydatidosis of the rib-case report and review of the literature. Ann Thorac Cardiovasc Surg.

[14] Traibi A, Atoini F, Zidane A (2010). Mediastinal hydatid cyst. J Chin Med Assoc.

[15] Sebit S, Tunc H, Gorur R (2005). the evaluation of 13 patients with intrathoracic extra pulmonary hydatidosis. J Int Med Res.

